# Crack formation and prevention in colloidal drops

**DOI:** 10.1038/srep13166

**Published:** 2015-08-17

**Authors:** Jin Young Kim, Kun Cho, Seul-a Ryu, So Youn Kim, Byung Mook Weon

**Affiliations:** 1 Soft Matter Physics Laboratory, School of Advanced Materials Science and Engineering, SKKU Advanced Institute of Nanotechnology (SAINT), Sungkyunkwan University, Suwon 440-746, Korea; 2 School of Energy and Chemical Engineering, Ulsan National Institute of Science and Technology (UNIST), Ulsan 689-798, Korea

## Abstract

Crack formation is a frequent result of residual stress release from colloidal films made by the evaporation of colloidal droplets containing nanoparticles. Crack prevention is a significant task in industrial applications such as painting and inkjet printing with colloidal nanoparticles. Here, we illustrate how colloidal drops evaporate and how crack generation is dependent on the particle size and initial volume fraction, through direct visualization of the individual colloids with confocal laser microscopy. To prevent crack formation, we suggest use of a versatile method to control the colloid-polymer interactions by mixing a nonadsorbing polymer with the colloidal suspension, which is known to drive gelation of the particles with short-range attraction. Gelation-driven crack prevention is a feasible and simple method to obtain crack-free, uniform coatings through drying-mediated assembly of colloidal nanoparticles.

Colloidal suspensions, where colloids or nanoparticles are uniformly suspended in a solvent, are widely used in industry. A drying process is usually adopted to deposit the colloids on a solid surface, allowing fabrication of thin colloidal films^1^. Drying-mediated assembly of colloidal nanoparticles^2^ is a cutting-edge technology. However, cracking of the dried colloidal films frequently takes place, particularly for those made from nanoparticle suspensions^3^,^4^,^5^,^6^,^7^,^8^,^9^,^10^,^11^,^12^, which can thus cause critical problems for application. Prevention of cracking is a significant task to improve the quality of colloidal films containing nanoparticles^13^,^14^, as well as to increase the applicability of large-area, highly ordered, and crack-free colloidal films^15^.

A variety of feasible methods for crack prevention have been suggested to date. For example, avoidance of cracking had been achieved through use of subsequent depositions of thin crack-free nanoparticle layers^13^, addition of hydrogels to suspensions to reduce their capillary pressures^16^, variation of the pH^17^ or addition of inorganic particles^18^ to control suspension flocculation, addition of a sol-gel glue^19^ or a sol-gel precursor^15^, addition of emulsion droplets to modulate suspension viscosity^14^, and use of organic colloids^20^ to enhance the fracture resistance of sol-gel coatings. Some examples of polymer addition for prevention of cracks can also be found in ceramic materials (for review, see^21^): polymers are added as binders into clays to increase fracture resistance^22^, which is an industrial tradition^23^, while poly(vinylalchohol) was found to reduce cracking in colloidal alumina^24^, and a variety of soft components, including polymers, soft spheres, and glycerol, were reported to increase fracture resistance in mixtures with colloids^25^. However, despite the many attempts which have been made to prevent cracking, a simple and highly versatile method that utilizes well-known and well-controlled physics, such as gelation, is still required to allow more effective elimination of cracking in various colloidal suspensions.

In principle, capillary pressures created by liquid menisci between colloidal particles are responsible for cracking^3^,^4^. While drying under ambient conditions, the capillary stresses normal to a colloidal film generate tensile stresses in the plane of the film^26^. From competition between the capillary and tensile stresses, the critical thickness of the film at which cracking would be initiated could be derived by balancing the critical stress for nucleation of an isolated crack and the Griffith’s criterion for equilibrium crack propagation^26^. The critical cracking thickness (CCT = *h*_*max*_) is determined by the maximum capillary pressure *P*_*max*_ beyond which the liquid menisci recede into the porous colloidal film, limiting deformation of the film^5^. The CCT is dependent on strain for soft colloids, which are deformable, while it exhibits stress-dependency for hard colloids which are very stiff and have negligible particle deformation^5^. Specifically, the CCT of hard colloids is dependent on the particle radius *r* and the particle shear modulus *G*. The *h*_*max*_ increases proportionally to *r* and *G*, as *h*_*max*_ ∝ *r*^3/2^*G*^1/2^, where 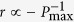
5,17. In practice, the film thickness, *h*, would depend on the initial particle volume fraction as well as the particle size. Therefore, the criterion of *h* > *h*_*max*_ for crack formation must be dependent on the initial particle volume fraction. In addition, *h* is a function of the droplet geometry during evaporation^27^,^28^. To prevent cracking at some conditions of *h* < *h*_*max*_, we need to have a detailed understanding of how colloidal drops evaporate, and how the generation of cracks depends on the particle size and the initial particle volume fraction.

The direct visualization of individual colloids inside drying colloidal drops would be helpful to uncover the details of crack formation for use in prevention. Herein, confocal laser microscopy was utilized to directly visualize the colloids during evaporation. By differences in contrast among the colloid, liquid, air, and their mixture^29^, how cracks are generated during evaporation can be determined, with examination of the dependency on particle size and initial volume fraction. As a versatile method for crack prevention, we attempted to drive gelation through addition of a nonadsorbing polymer to the colloidal suspension, following the previously developed method^30^. The results demonstrated that this approach driving weak gelation with short-range attraction^30^ is feasible for the fabrication of uniform, crack-free coatings from colloidal drops. This approach will likely be useful in the fabrication of uniform, crack-free colloidal films for applications associated with inkjet printing, paints, coatings, and ceramics.

## Results

### Evaporation complexity of colloidal droplets

**Figure 1:**
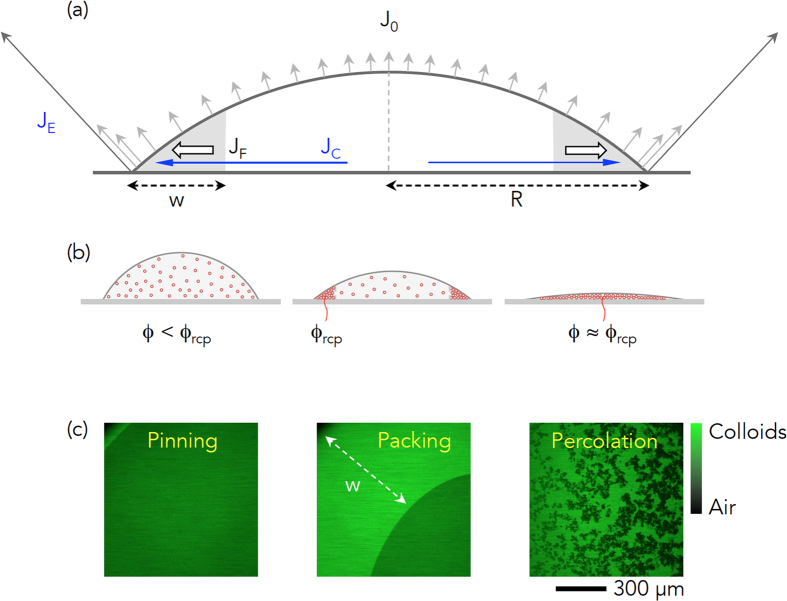
Evaporation complexity of colloidal droplets. (**a**) Hydrodynamic flows during evaporation: the evaporative vapor flux, *J*_*E*_, from the droplet surface to the atmosphere and *J*_0_ at the center; the outward coffee-ring flow, *J*_*C*_, induced by *J*_*E*_; and the fluid flow, *J*_*F*_, through the compact region with a width, *w*, as marked by the gray part. (**b**) Three evaporation stages with respect to the growth of the compaction region with width *w* up to a final width, *w*_*f*_: *w* ≈ 0 at the initial (pinning) stage, 0 < *w* < *w*_*f*_ at the intermediate (packing) stage, and *w* ≈ *w*_*f*_ at the final (percolation) stage. Through evaporation, the particle volume fraction *ϕ* reached the random close packing, *ϕ*_*rcp*_. (**c**) Confocal microscopic observations for each stage, determined by differences in fluorescence intensities among the air, solvent, and colloids.

The complicated dynamics involved in the evaporation of colloidal droplets are schematically illustrated in Fig. 1. The complexity of droplet evaporation has been widely studied since the discovery of the coffee-ring effect^31^ (see^1^ for a recent review). When a spherical cap drop evaporates on a flat solid surface in still air, the following three hydrodynamic flows are generated inside the droplet, influencing the final deposition patterns after evaporation (Fig. 1a): the evaporative vapor flux, *J*_*E*_, from the droplet surface to the atmosphere and *J*_0_ at the droplet center; the outward coffee-ring flow, *J*_*C*_, induced by *J*_*E*_; and the fluid flow, *J*_*F*_, traveling through the compact region with a width, *w*, marked by the gray part in Fig. 1a [Ref. 3]. Consequently the *outward* flows, *J*_*C*_, by the coffee-ring effect^31^ and *J*_*F*_ by Darcy’s law^3^,^32^, influence the drying-mediated compaction dynamics through solute accumulation toward the droplet edge.

The complicated evaporation dynamics can be considered to occur in three stages, as illustrated in Fig. 1b, with respect to growth of the compaction region with a width, *w*. At the initial (pinning) stage, *w* ≈ 0, where the colloidal particles are evenly distributed throughout a droplet with radius *R*, where only a few particles can induce *self-pinning* as a requisite for the coffee-ring effect^33^. At the intermediate (packing) stage, 0 < *w* < *w*_*f*_ where the particles are packed at the edge. The dense edge may reach a random close packing *ϕ*_*rcp*_^34^ and gradually grow toward the center up to a final width *w*_*f*_^35^. At the final (percolation) stage, *w* ≈ *w*_*f*_ where the compaction region occupies the entire part. The particle volume fraction reaches *ϕ*_*rcp*_ and the surrounding air starts to replace the solvent. Depending on the particle size, air can invade by percolation or bursting for large particles, as well as by cracking for small particles^35^.

The three evaporation stages could clearly be identified through confocal laser microscopy and use of fluorescent-dyed colloidal particles, as demonstrated in Fig. 1c. In particular, each stage was distinguishable owing to differences in the fluorescence intensities among the air, solvent, and colloids. Confocal microscopy has widely been used for the direct observation of real-space motions of individual colloids^29^. A confocal laser microscope is a laser scanning optical microscope that utilizes a fluorescent technique^36^. With this approach, we are able to directly investigate crack initiation and growth dynamics. A similar approach was used in recent reports for crack studies^14^,^37^. Confocal microscopy is also useful for studies on evaporation gradients^38^, as well as on evaporative lithography^39^.

### Crack initiation and growth

**Figure 2:**
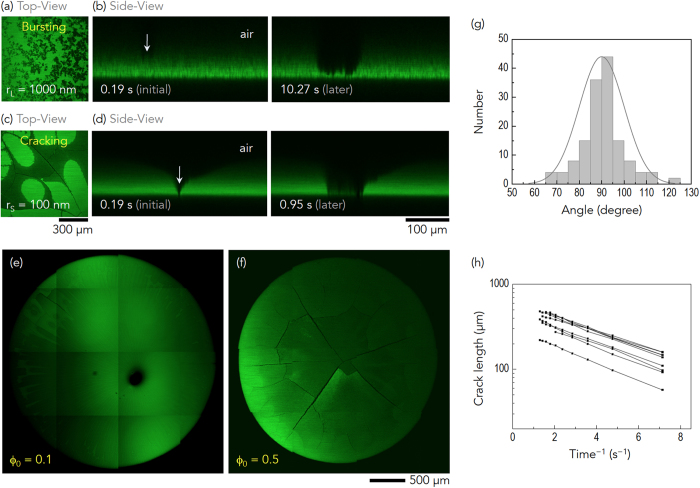
Crack initiation and growth. (**a**–**d**) The particle size effect for samples with the same 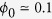 for different radii: *r*_*L*_ = 1000 nm and *r*_*S*_ = 100 nm. The top-view and the side-view confocal images for large [small] colloids (**a**,**b**) [(**c**,**d**)], respectively, suggest that air invasion took place via bursting for large colloids and via cracking for small colloids. (**e**,**f**) The initial particle volume fraction effect with the same *r*_*S*_ = 100 nm, with variation of 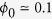 and 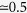, showed that the cracks become thick and their spacing become wide at high *ϕ*_0_. A radial spreading of cracks is demonstrated in (**f**) and Supplementary Movie 1. (**g**,**h**) The crack growth dynamics: right angles between cracks are frequent in (**g**) and the crack length *L* grows with time t in (**h**), showing proportionality as *L* *~* exp(*A* + *B*/*t*) where *A* and *B* are constants.

To gain an understanding of the crack formation mechanism during evaporation of colloidal droplets, the particle size effect was first examined, as in Fig. 2a–d. Identical colloidal droplets containing hard sphere-like PMMA colloids with the same initial particle volume fraction of 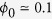 were utilized, except the particle radius was set to *r*_*S*_ = 100 nm for small colloids and to *r*_*L*_ = 1000 nm for large colloids. Here, it could be observed that the air invasion at the final evaporation stage actually took place via *cracking* for small colloids and *bursting* for large colloids. The top-view confocal images for large colloids (Fig. 2a) and small colloids (Fig. 2c) confirmed that cracking would be generated more favorably in the colloidal droplets with smaller colloids. The side-view confocal images for large colloids (Fig. 2b) and small colloids (Fig. 2d) demonstrated that the air invasion would be more rapid via cracking than bursting at the same thickness, *h* ~ 100 *μ*m. In principle, the critical cracking thickness, *h*_*max*_, is proportionally dependent on the particle radius, *r*, as *h*_*max*_ ∝ *r*^3/2^ [Ref. 5, 17]. Our observations were consistent with the known crack formation mechanism, which cracking is generated more frequently for smaller colloids at a fixed coating thickness.

Next, the particle volume fraction effect was examined using identical colloidal droplets for small colloids *r*_*S*_ = 100 nm with different *ϕ*_0_ as 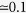 (Fig. 2e) and 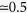 (Fig. 2f). Upon examination, the cracks were observed to become thicker with wider spacing at high *ϕ*_0_, which is consistent with other observations^40^. This would be associated with the final thickness, as well as the initial particle volume fraction. Interestingly, at high *ϕ*_0_, the thickness would be maximized at the droplet center, thus the cracking would initiate from the center. Accordingly, radial spreading of the cracks could be observed in Fig. 2f (Supplementary Movie 1).

Finally, the crack growth dynamics were considered. From various confocal movies, we measured the angles between cracks, as summarized in Fig. 2g, which revealed the most frequent angles to be 90 degrees. This suggests that the crack growth was associated with the propagation-dominated mechanism, which is frequently observed in desiccation crack networks^41^. Most interestingly, measurement of the crack length, *L*, as a function of time, *t*, in Fig. 2h, revealed proportionality as 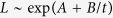, equivalent to ln *L* ∝ 1/*t*, where *A* and *B* are constants. Such crack growth dynamics have not yet been reported elsewhere. This proportionality indicates that the crack growth accelerates over time, similar to the Arrhenius relation, where a reaction rate is increased at higher temperature.

Crack initiation and growth are inevitable problems for small colloids: therefore, an effective method should be developed to prevent cracking of dried colloidal drops with small colloids.

### Crack prevention with gelation

To prevent the intrinsic crack formation during the evaporation of colloidal drops with small colloids, we examined a versatile method utilizing gelation through addition of a nonadsorbing polymer. Mixing a nonadsorbing polymer into a colloidal suspension is known to drive colloidal aggregation or gelation with short-range attraction^42^. To demonstrate the effectiveness of the gelation-mediated crack prevention, the well-studied nonadsorbing linear polystyrene (PS) depletant polymer (Polymer Labs, *M*_*w*_ = 6.67 × 10^5^, *M*_*w*_/*M*_*n*_ = 1.03 [the weight average molecular weight versus the number average molecular weight]) was added to the colloidal suspension with small colloids.

**Figure 3:**
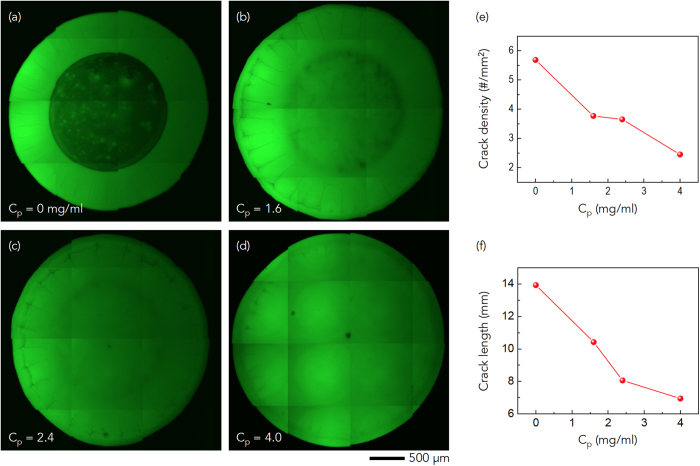
Gelation-mediated crack prevention. (**a**–**d**) The addition of the nonadsorbing PS polymer effectively prevented crack formation in the dried colloidal drops with *r*_*S*_ ~ 100 nm, *ϕ*_0_ ~ 0.1, and *R*_0_ ~ 1.4 mm, as the concentration increased from *C*_*p*_ = 0 to 4.0 mg/ml. In particular, almost all cracks were removed in (**d**), for *C*_*p*_ = 4.0 mg/ml (Supplementary Movie 2). The total crack density (**e**) and the total crack length (**f**), determined from (**a**–**d**), provide quantitative evidence for the gelation-mediated crack prevention.

The free-volume PS concentration *C*_*p*_ (=mg/ml) was controlled in a suspension with *r*_*S*_ ~ 100 nm, *ϕ*_0_ ~ 0.1, and *R*_0_ ~ 1.4 mm (=the initial drop radius). As illustrated in Fig. 3, the addition of the nonadsorbing PS polymer effectively prevented crack formation in the dried colloidal drops as the concentration increased from *C*_*p*_ = 0 to 4.0 mg/ml. Time-resolved tile scans obtained via confocal microscopy were utilized for crack characterization and visualization of the crack prevention. It was found that the cracks formed along the ring-like deposits in Fig. 3a could be effectively eliminated by the addition of the PS polymer, with reduction of the cracks manifested in Fig. 3b–d. In particular, almost all cracks had disappeared in Fig. 3d for *C*_*p*_ = 4.0 mg/ml (Supplementary Movie 2). The total crack density (Fig. 3e) and total crack length (Fig. 3f) determined from Fig. 3a–d demonstrate the significant prevention of crack formation by addition of the PS polymer. Interestingly, the coating also became more uniform as the amount of PS polymer increased. This suggests that the gelation-mediated crack prevention would be a feasible method for fabricating uniform, crack-free colloidal films through drying-mediated assembly of colloidal nanoparticles.

## Discussion

The addition of the PS polymer to a suspension can induce a depletion attraction between colloids, thus causing the *gelation* phenomenon. Gelation would *retard* the compaction dynamics at the droplet edge, in the presence of the intrinsic *outward* hydrodynamic flows. It can be considered that the initial gelation dynamics should be fast enough to retard the particle compaction dynamics at the droplet edge.

**Figure 4:**
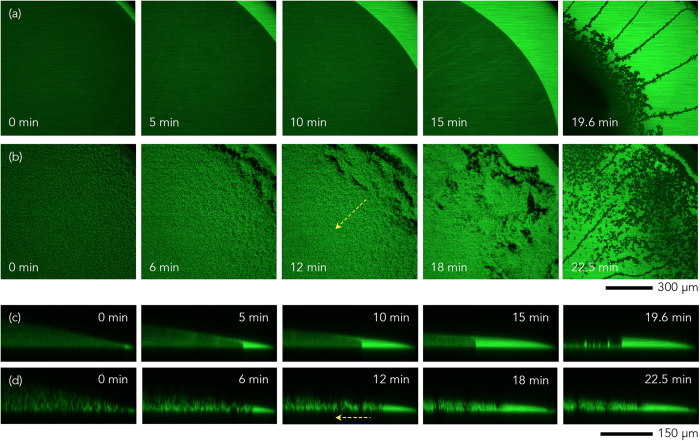
Temporal evolution of gelation. (**a**) The typical coffee-ring effect appeared in the colloidal suspension drops (Supplementary Movie 3), identical to the sample of Fig. 3a, (**b**) while the gelation by addition of polymer at *C*_*p*_ = 4.0 mg/ml (Supplementary Movie 4), identical to the sample of Fig. 3d, displayed significant retardation of the outward coffee-ring effect (by the arrows). This result was repeated in the side-view sequential confocal images without the polymer addition in (**c**) (Supplementary Movie 5) and with polymer addition in (**d**) (Supplementary Movie 6).

The top-view sequential confocal images in Fig. 4 provide direct evidence of the gelation-induced *reverse* coffee-ring effect. The typical coffee-ring effect appeared in the colloidal suspension drop, identical to the sample of Fig. 3a, as seen in Fig. 4a (Supplementary Movie 3), while gelation with the addition of polymer at *C*_*p*_ = 4.0 mg/ml in Fig. 4b (Supplementary Movie 4) significantly retarded the outward coffee-ring effect. This result was repeated in the side-view sequential confocal images without polymer addition in Fig. 4c (Supplementary Movie 5) and with polymer addition in Fig. 4d (Supplementary Movie 6).

**Figure 5:**
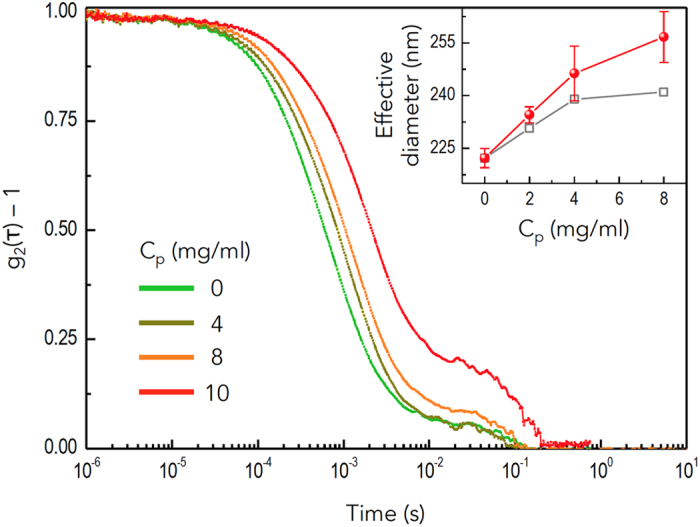
Light scattering measurements. Diffusing wave spectroscopy (DWS) and dynamic light scattering (DLS) were adopted to show the onset of gelation. (main) The DWS data, expressed as the intensity autocorrelation function *g*_2_(*τ*) − 1 versus time, show that the relaxation dynamics became slower, as *C*_*p*_ increased, indicating the onset of gelation. (inset) The effective diameter, taken from the intensity average diameter of the DLS data (circles), increased with *C*_*p*_, even considering the estimated viscosity increment (squares). The occurrence of gelation at *C*_*p*_ = 4 mg/ml was consistent with the experimentally measured gelation boundary of 3.7 or 3.25 mg/ml reported in the literature.

To verify the onset of gelation in the suspensions containing PS polymer, light scattering experiments were conducted. Diffusing wave spectroscopy (DWS) experiments were performed to examine the changes of the autocorrelation functions of the interacting particles, given by 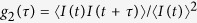, where *I*(*t*) is the light intensity and 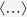 denotes the ensemble averaging^43^,^44^. Figure 5 shows the intensity autocorrelation function, *g*_2_(*τ*) − 1, for varying polymer concentrations. The autocorrelation function of particles with no polymer approximately displayed an exponential decay mainly associated with the fast relaxation process of the particles within the cages formed by their neighbors, often called the *β* process. As the polymer concentration increased, the autocorrelation decayed more slowly. The slowed relaxation of the particle dynamics can be attributed to the aggregation of particles or formation of clusters, noting that particles experience gelation over a long time period. The second relaxation process would be associated with the cooperative particles, often called the *α* process. (The detailed analysis of the *α* and *β* processes will be discussed elsewhere.) Dynamic light scattering (DLS) experiments were also performed to measure the hydrodynamic sizes of the particles in the dilute particle limit at each polymer concentration^45^. The DLS results, shown in the inset of Fig. 5, revealed that the effective diameter of the colloids increased upon addition of the PS polymer. The PMMA colloids had the radius of *r* ≈ 111 ± 1.4 nm, which fits well with the size reported by the manufacturer. As polymers were added, the particles started to show a concentration-dependent increment of the size with increasing polydispersity. Particles in these experiments displayed *ξ* = *r*_*p*_/*r* ~ 0.11 at *C*_*p*_ = 4 mg/ml well above the experimentally determined gelation boundary 
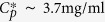
46 or 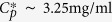
30 at 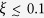 and *ϕ*_0_ ~ 0.1. The increase in the effective diameter is evident, even considering the estimated viscosity increment^47^. Therefore, the increased particle size was attributed to the average size of the particle clusters formed. Both light scattering experiments supported the gelation of particles, determining that the particles experienced net attractions. In addition, the particle compaction process at the droplet edge during evaporation (Fig. 4) can lessen the 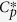 required for gelation^46^. The above evidence confirms that the gelation dynamics near the droplet edge can effectively retard the outward coffee-ring flows.

**Figure 6:**
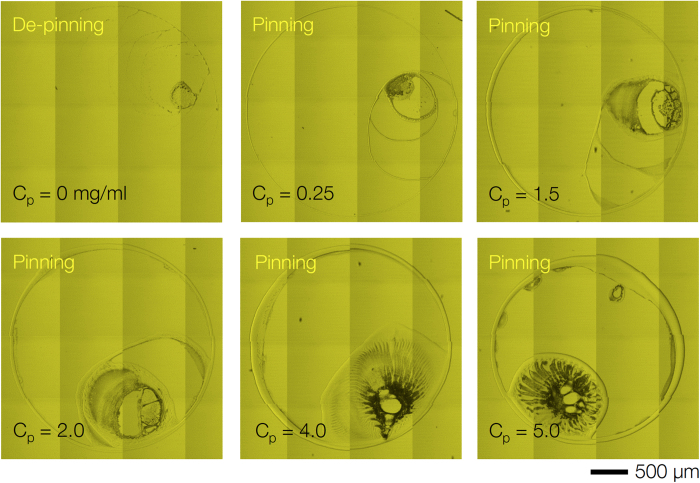
De-pinning and pinning at polymer suspended droplets. Without the colloidal particles, the suspension including decalin solvent and PS polymer showed typical de-pinning at *C*_*p*_ = 0 and the pinning behaviors at *C*_*p*_ > 0, which is irrelevant to crack formation.

It should be noted that the suspension including only the decalin solvent and the PS polymer, without colloids, displayed the typical de-pinning behavior at *C*_*p*_ = 0 and pinning behavior at *C*_*p*_ > 0, as shown in Fig. 6. The ring width became larger as the polymer concentration increased. This suggests that the addition of the polymer into the solvent without colloidal particles had no effect on the crack formation. The pinning behavior of pure polymer solutions may show somewhat exotic patterns after evaporation^48^.

In conclusion, we demonstrated that cracking is an intrinsic and inevitable phenomenon for colloidal films made with hard sphere-like small colloids or nanoparticles (with radii at the nanoscale). We directly visualized the generation of cracks during evaporation of colloidal droplets using confocal laser microscopy and standard colloidal suspensions, with fluorescent-dyed colloids. From direct tracking of the individual colloids via confocal microscopy, we confirmed the critical effects of the particle size and initial concentration on crack formation. For versatile crack prevention, we demonstrated the gelation-mediated crack prevention that enabled us to obtain uniform, crack-free coatings through drying-mediated assembly of colloidal nanoparticles. This crack prevention method would be useful to obtain crack-free uniform colloidal films for inkjet printing, paints, coatings, and ceramics.

## Methods

### Materials

For direct tracking of individual particle motions with confocal laser microscopy, poly(methyl methacrylate) (PMMA) colloidal particles labeled with a fluorescent dye were used^49^,^50^. As standard solvents for the colloidal droplets, a mixture of cis- and trans-decalin (Decahydronaphthalene, 99%, Sigma-Aldrich), were employed which has widely been used in drying experiments^49^,^51^,^52^. Each droplet was gently deposited onto a clean cover glass (VWR, 22 × 30 mm^2^, No. 1.5) in all experiments. The initial drop volume was controlled to be *V*_0_ ≈ 0.5 *μ*l, resulting in the initial contact radius of *R*_0_ ≈ 1.4 mm, which is smaller than the capillary length (≈1.9 mm for decalin): therefore, the droplet shape was assumed to be spherical. The PMMA colloids were prepared and supplied by A. Schofield (University of Edinburgh) according to the procedure described by Antl *et al.*^53^, and thus were expected to show hard-sphere-like behaviors. The colloid radii were *r*_*S*_ = 100 nm (small colloids) and *r*_*L*_ = 1000 nm (large colloids) with ~5% polydispersity in size (previously determined by dynamic light scattering^33^). The density difference between colloids (1.19 g/cm^3^) and decalin (0.897 g/cm^3^) was small enough to prevent any sedimentation issues for the microscopic colloids^54^.

### Confocal microscopy

Real-time direct observation of colloidal particles inside drying droplets was carried out using confocal laser microscopy (Leica TCS SP5). The XY or XZ scans for top-view or side-view imaging were utilized in Figs 1c, 2a–d and 4a–d. Particularly based on the confocal movies, characterization of the cracks, such as the crack angle distribution and the crack growth dynamics, were carried out in Fig. 2g-h. For the time-resolved scans, the XYT tile scans with confocal microscopy were performed, as shown in Figs 2e-f, 3a–d, and 6.

### Crack prevention with gelation

To achieve gelation-mediated crack prevention, a nonadsorbing linear polystyrene depletant polymer (Polymer Labs, *ξ* ~ 0.11, *M*_*w*_ = 6.67 × 10^5^, and *M*_*w*_/*M*_*n*_ = 1.03) was added to PHSA-stabilized PMMA colloidal suspensions. This polymer has been well studied for induction of depletion attraction between the PMMA spheres at 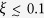 and *ϕ*_0_ ~ 0.1. Variation of the free-volume concentration, *C*_*p*_ (=mg PS per ml of solvent), was also carried out, as shown in Figs 3 and 6.

### Light scattering experiments

DWS experiments in Fig. 5 were performed on a DWS RheoLab II (LS Instruments) in transmission mode to obtain the autocorrelation function of the dense particle systems. The coherent source was a diode laser (*λ* = 658 nm; 30 mW). The samples were measured at 25 ± 0.02 °C. DLS experiments in Fig. 5 were performed with the BI-200 SM goniometer (Brookhaven Instruments) using an EM-9865 photomultiplier and a digital correlator (BI-9000AT) for measurement of the intensity average diameters of particles with varying polymer concentrations.

## Additional Information

**How to cite this article**: Kim, J. Y. *et al.* Crack formation and prevention in colloidal drops. *Sci. Rep.* **5**, 13166; doi: 10.1038/srep13166 (2015).

## Supplementary Material

Supplementary Information

Supplementary Movie 1

Supplementary Movie 2

Supplementary Movie 3

Supplementary Movie 4

Supplementary Movie 5

Supplementary Movie 6
