# Supplementary material for: Crack formation and prevention in colloidal drops
Source: Sci Rep. 2015 Aug 17;5:13166. doi: 10.1038/srep13166 (PMC4538394; doi:10.1038/srep13166)
Supplement: Supplementary Information [file srep13166-s1.pdf]

Scientific Reports

Supplementary Information

Crack formation and prevention in colloidal drops

Jin Young Kim, Kun Cho, Seul-a Ryu, So Youn Kim, and Byung Mook Weon*

Brief explanations for supplementary movies:

Name	Snapshot	Comments
Supplementary Movie 1	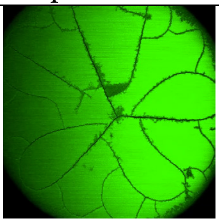	Confocal movie for Fig. 2f.
Supplementary Movie 2	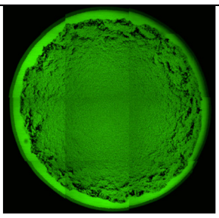	Confocal movie for Fig. 3d.
Supplementary Movie 3	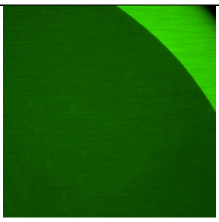	Confocal movie for Fig. 4a.
Supplementary Movie 4	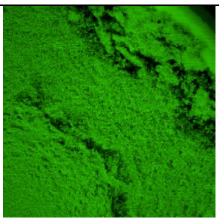	Confocal movie for Fig. 4b.
Supplementary Movie 5	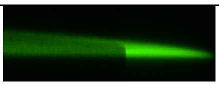	Confocal movie for Fig. 4c.
Supplementary Movie 6	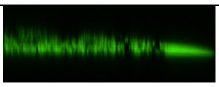	Confocal movie for Fig. 4d.

*E-mail: bmweon@skku.edu
